# The Role of the Vagus Nerve: Modulation of the Inflammatory Reaction in Murine Polymicrobial Sepsis

**DOI:** 10.1155/2012/467620

**Published:** 2012-03-21

**Authors:** Wolfram Kessler, Stephan Diedrich, Pia Menges, Tobias Ebker, Michael Nielson, Lars Ivo Partecke, Tobias Traeger, Katharina Cziupka, Julia van der Linde, Ralf Puls, Alexandra Busemann, Claus-Dieter Heidecke, Stefan Maier

**Affiliations:** ^1^Klinik und Poliklinik für Allgemeine Chirurgie, Viszeral, Thorax und Gefäßchirurgie der Ernst Moritz Arndt Universität Greifswald, Friedrich Loeffler Straße 23b, 17475 Greifswald, Germany; ^2^Mund, Kiefer und Gesichtschirurgische Klinik der Universität Erlangen, Glückstraße 11, 91054 Erlangen, Germany; ^3^Institut für diagnostische Radiologie und Neuroradiologie der Ernst Moritz Arndt Universität Greifswald, Ferdinand-Sauerbruch-Straße, 17475 Greifswald, Germany

## Abstract

The particular importance of the vagus nerve for the pathophysiology of peritonitis becomes more and more apparent. In this work we provide evidence for the vagal modulation of inflammation in the murine model of colon ascendens stent peritonitis (CASP). Vagotomy significantly increases mortality in polymicrobial sepsis. This effect is not accounted for by the dilatation of gastric volume following vagotomy. As the stimulation of cholinergic receptors by nicotine has no therapeutic effect, the lack of nicotine is also not the reason for the reduced survival rate. In fact, increased septic mortality is a consequence of the absent modulating influence of the vagus nerve on the immune system: we detected significantly elevated serum corticosterone levels in vagotomised mice 24 h following CASP and a decreased *ex vivo* TNF-alpha secretion of Kupffer cells upon stimulation with LPS. In conclusion, the vagus nerve has a modulating influence in polymicrobial sepsis by attenuating the immune dysregulation.

## 1. Introduction

Peritonitis and subsequent sepsis remain a severe problem in the surgical field. The immune response of a septic organism is mediated by the adaptive and the innate immune system. Beside “classical” mechanisms like humoral and paracrine regulation or direct cell-to-cell interaction, the autonomic nervous system seems to be critically involved. Neural circuits that directly control certain immune reactions, also known as the “inflammatory reflex”, were identified [1–3]. Within these neural circuits, the vagus nerve plays an important role as it mediates afferent effects triggered by inflammatory mediators and also interacts with visceral organs by efferent activity.

During sepsis, the vagus nerve is essential for balancing anti- and pro-inflammation. Experimental vagotomy results in hyperinflammation and can lead to increased mortality [4–7]. Several studies showed that animals deficient in vagus nerve activity are more sensitive to inflammatory challenges like endotoxemia, sepsis, pancreatitis [8] and hypovolemic shock. This may be due to the uncontrolled hyperinflammation which is mirrored by a critically increased release of proinflammatory cytokines [1, 6, 7, 9].

An efferent vagal connection to adrenal glands is established [10–12]. However, there is still an incomplete understanding of the potential role of vagal nerve in context of adrenal gland derived glucocorticoids. It is known that adrenal gland function is essential for the outcome of patients with sepsis [13–15]. Nevertheless, glucocorticoid therapy in sepsis, based on their ability to induce IL-10 elevation und TNF-alpha decrease, was controversially discussed [16–18]. Additionally, vagal activity seems to be involved in fever regulation [19, 20], apoptosis [21, 22], and regeneration of hepatocytes [23]. The liver and its resident macrophages, the Kupffer cells (KCs), are considered to play a crucial role in the course of sepsis [24]. An anatomical link between the vagus nerve and the liver is well described: the hepatic branch of the vagus nerve [25, 26]. KCs produce many kinds of soluble mediators such as cyokines, especially IL-10 and TNF-alpha, prostanoids, proteases, and oxygen radicals [27, 28]. 80–90% of them are located in the liver [27]. A vagal modulation of the cytokine release by Kupffer cells in inflammation is presumed, but poorly understood [29].

The aim of this study was to further investigate the role of the vagus nerve in the course of peritonitis. Therefore, the colon ascendens stent peritonitis (CASP) was used. In contrast to models of LPS shock, CASP is a model of polymicrobial abdominal sepsis, that mirrors a common course of systemic infection in surgical intensive care patients [30–32]. The influence of subdiaphragmal vagotomy on corticosterone release by adrenal glands and KCs function after CASP were examined. Additionally, the potential therapeutic effect of nicotine as an unspecific agonist of nicotinic acetylcholine receptors (nACHRs) during peritonitis was analyzed.

## 2. Methods

### 2.1. Mice

For all experiments, 8- to 12-week-old female C57BL/6 mice purchased from Charles River (Sulzfeld, Germany) (weight 20–25 g) were used. Prior to surgery, mice were kept for at least 2 weeks in the animal facility to recover from transport. All experimental procedures were performed according to German animal safety regulations. For all surgical procedures, Avertin (Sigma-Aldrich Chemie, Taufkirchen) anaesthesia was used.

### 2.2. CASP Surgery

The surgical procedure for CASP was performed as previously described [30, 31]. After disinfection of the abdomen, the ascending colon was identified and a prepared catheter (16 gauge, Venflon; BOC, Ohmeda, Sweden) was implanted in the antimesenteric wall of the ascending colon. To ensure intraluminal positioning of the stent, stool was milked from the ascending colon into the stent. Afterwards, 0.5 mL of sterile saline solution was flushed into the peritoneal cavity before closure of the abdominal walls (single layer; 4/0 Polyester, Catgut, Markneukirchen, Germany).

### 2.3. Vagotomy

**Figure 1:**
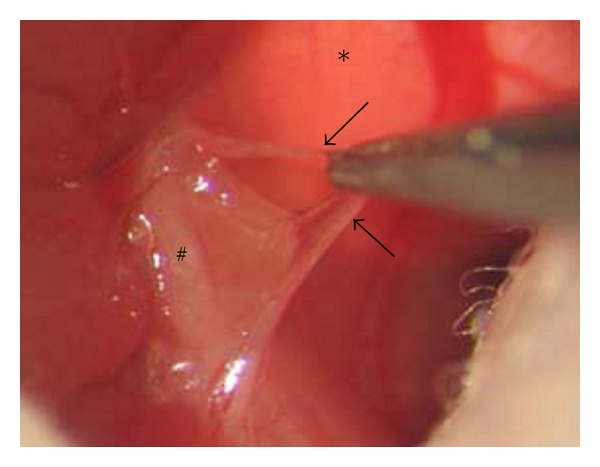
Surgical dissection of the vagus nerve using a microscope (40-fold magnification): the abdominal wall was opened through a transverse incision. Esophagus (#) and diaphragm (∗) were exposed and the ventral branch of the vagal nerve (arrows) was dissected. The dorsal branch of the vagus nerve was excised in the same way.

Upper abdominal wall was opened through a transverse incision. Esophagus was exposed by carefully keeping costal arc, liver, and stomach out of sight. Further preparation was done using a surgical microscope (40-times magnification, Leica M651, Bensheim, Germany). The ventral branch of the vagal nerve was exposed and about 3 mm were excised (see Figure 1). After its passage through the diaphragm, the esophagus was mobilized on its hepatic side and lifted. The dorsal branch of the vagal nerve was exposed and about 3–5 mm were resected. After fluid resuscitation (0.5 mL of sterile saline solution), the abdominal wall was closed (one layer; 4/0 Polyester, Catgut, Germany). For control purposes, sham operations without transsection of the vagal nerve were performed.

### 2.4. Implantation of Osmotic Pumps

Alzet osmotic pumps (Modell 1002, Alzet, Cupertino, USA) where filled with nicotine dissolved in 0.9% NaCl according to manufacturer instructions. With a liberation volume of 0.20–0.22 *μ*L/h nicotine concentrations were adapted to the individual weight of each animal to ensure the expected liberation rate of 1, 1.5, and 3 mg/kg bodyweight (BW)/h. Control groups received osmotic pumps filled with 0.9% NaCl. After a short incision in the neck region of the mice, the primed pumps were placed in a subcutaneous pouch. Thereafter, the incision was closed (4/0 Polyester, Catgut, Germany).

### 2.5. Isolation of Kupffer Cells

The isolation procedure was modified from Valatas et al. [33]. All steps were performed with sterile solutions at pH = 7.4. The portal vein was identified, punctured with a 22-gauge cannula and irrigated by 50 mL pretempered HBSS. To allow the irrigation solution to evade the venous system, the inferior vena cava was incised right above its bifurcation. Afterwards, the liver was perfused with pronase E 0.4% for enzymatic digestion of hepatocytes and connective tissue and disintegration of cell junctions. A second enzyme mixture containing collagenase 0.0143% and DNAse 0.0014% was applied to dissolve the extracellular matrix and released DNA. The liver was transferred onto a petri dish, the capsule was cut and the parenchyma carefully fragmented before a third enzymatic digestion (using pronase E 0.1% and DNAse 0.01%) was performed under constant agitation. The cell suspension was pushed through a nylon mesh to remove larger cell aggregates. Subsequent steps were conducted with 4°C media to abate cell attachment on plastic surfaces.

A step of differential centrifugation followed (10 min, 380 g, 4°C) to wash out the residual enzymatic solution, DNA, and cell debris. The supernatant was discarded, the pellet resuspended and density gradient centrifugation was performed to separate parenchymal from nonparenchymal cells. Iodixanol (OptiPrep) was applied as seperation medium. HBSS and OptiPrep were added to the cell suspension to gain 4 mL of a 11.7% OptiPrep solution which was bedded on a density cushion of 4 mL 17.6% OptiPrep. An additional 4 mL of HBSS was used as a top finishing. Density centrifugation was carried out at 1400 g and 4°C for 17 min. The resulting layer of mainly non-parenchymal cells on top of the 11.7% OptiPrep cushion was carefully removed and transferred into RPMI+ medium followed by another centrifugation step (10 min, 380 g, 4°C). The pellet was resuspended with 1 mL RPMI+.

Total number of non-parenchymal cells was assed using the Neubauer chamber. Adding the appropriate amount of RPMI+ a final cell concentration of 5 × 10^4^/mL was generated. For cell culture a 96-well culture plate was used and 200 *μ*L of cell suspension was added. Kupffer cell function was shown by phagocytosis of fluorescing latex beads (3 *μ*m Fluoresbrite). Kupffer cell purity was analysed by cell adherence to glass slides and subsequent immunofluorescence staining with FITC-conjugated anti-F4/80 antibody. We reached purity of 79 percent. Cells were kept at 37°C and 5% CO_2_-atmosphere. After 24 hours of incubation, cell media and all nonadherent cells were removed by thorough washing. All subsequent stimulation experiments were performed using FCS-free media.

### 2.6. Stimulation of Kupffer Cells

Kupffer cells were cultured in 96-well plates containing 1 × 10^4^ cells per well in 200 *μ*L cell culture medium (RPMI without FCS). After 24 hours, medium was changed and cells were stimulated with LPS (*E. coli*, Sigma-Aldrich Chemie, Taufkirchen) at concentrations of 0.1 *μ*g/mL, 1 *μ*g/mL and 10 *μ*g/mL (same volume of medium in each well) dissolved in 1 × PBS. After 24 hours, the supernatant was transferred to 1.5 mL Eppendorf tubes and centrifuged (10 min; 16100 g; 4°C; Centrifuge 5415R, Eppendorf, Germany). Cytokines/chemokines were analysed using a commercial available kit (BD cytometric bead array mouse inflammation kit, BD bioscience, Heidelberg, Germany).

### 2.7. MRI Imaging and Analysis

MRI was modified from Partecke et al. [34]. For all MRI studies, anaesthesia had to be carried out using isoflurane (1%–1.5%). The depth of anaesthesia was monitored by the breathing rate (about 40 breaths per minute). MRI sequences were triggered by breathing rate. To reduce the influence of bowel motility in all MRI examinations, mice were kept *nil per os* (NPO) for at least 4 hours before starting MRI scans. For MRI scans, all mice were placed in a supine position. Mice were scanned in a high-field 7.0 Tesla MRI scanner for small animals (Bruker, ClinScan, 7.0 Tesla, 290 mTesla/m gradient strength, Bruker, Ettlingen, Germany). MRI scans were performed in a whole mouse body coil (Bruker, Ettlingen, Germany) using a T2-TSE (turbo spin echo) sequence. For size and volume assessment, we used high resolution coronary and axial T2-weighted images (coronary plane: TR (repetition time): ca. 1200 ms; TE (echo time): 41.0 ms; FA (flip angle): 180°; FoV (field of view): 42 mm × 42 mm; matrix: 240 × 320; 24 slices of 0,7 mm per slice, acquisition time: ca. 15 min; axial plane: TR: ca. 1250 ms; TE: 41.0 ms; FA: 180°; FoV: 40 mm × 40 mm; matrix: 240 × 320; 24 slices of 0,7 mm per slice, acquisition time: ca. 10 min). Generated images were analyzed using MIPAV (medical imaging processing and visualisation, National Institutes of Health, Bethesda, MD, USA) and Image J (Image Processing and Analysis in Java, National Institutes of Health). By defining regions of interest (ROI) on each slice, the software was able to calculate volumes and diameters. This was finally done by a complex algorithm using all image inherent information including thickness of slices, resolution as well as size of ROIs.

### 2.8. Serum Corticosterone Levels

For the detection of serum corticosterone levels, we used a commercially available ELISA-kit (Corticosterone (Rat/Mouse) Elisa, DRG Instruments GmbH, Marburg, Germany) following customers instructions. Serum was separated by centrifugation of whole blood (10 min; 16100 g; Centrifuge 5415R, Eppendorf, Germany).

### 2.9. Survival Analysis

Survival of animals was observed for 240 hours after CASP induction.

### 2.10. Statistical Methods

Statistical analysis was performed using GraphPad Prism for Windows software (GraphPad Software, San Diego, CA, USA). Statistical differences in survival rates were assessed using log-rank test. Results from cytokine levels were analyzed using the two-tailed Mann-Whitney *U* test for nonparametric probes. A significance level of 0.05 was applied for all calculations.

## 3. Results

### 3.1. Vagotomy Increases the Mortality in Polymicrobial Sepsis

**Figure 2:**
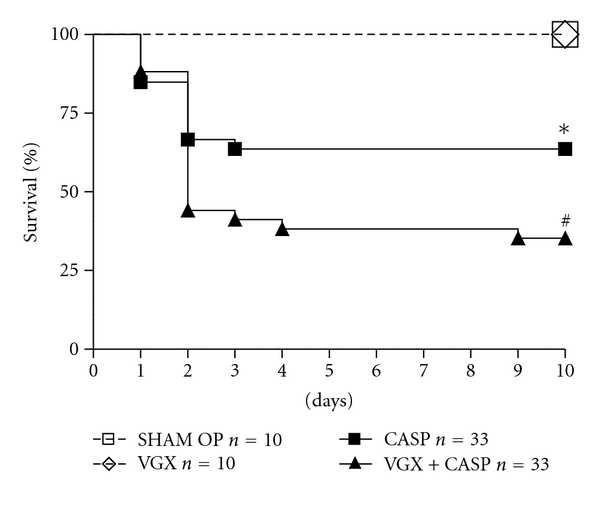
The mortality in polymicrobial sepsis (CASP) is significantly increased compared to sham-operation (laparotomy without CASP or vagotomy) by 63.6% versus 100% (*P = 0.025, *n* = 10 and 33, resp.). The survival of the vagotomised CASP group (VGX + CASP, *n* = 33) is significantly decreased further to 35.3% (CASP versus VGX + CASP: ^#^ *P* = 0.048). Vagotomy itself (*n* = 10) does not affect the survival rate (100%).

To analyse the influence of the vagus nerve on the mortality in polymicrobial sepsis, we compared the survival rate of mice in the following surgical procedures: CASP, vagotomy (VGX), CASP in combination with vagotomy. Sham-operation was performed in the control group. Sham-surgery as well as vagotomy did not change the survival rate of mice as both were 100% (Figure 2, *n* = 10 per group). Comparable to our recent data, the induction of a septic peritonitis by CASP significantly decreased the survival rate to 63.6% (*P* = 0.025, *n* = 33). The survival of the vagotomised CASP group (VGX + CASP, *n* = 33) was significantly decreased further to 35.3% (*P* = 0.048). Thus, the intact vagus function is essential for the survival in polymicrobial sepsis, whereas vagotomy in absence of a septic focus does not affect the survival rate.

### 3.2. The Effect of Enlarged Gastric Volume by Vagotomy Is Independent of the Presence of Sepsis

**Figure 3:**
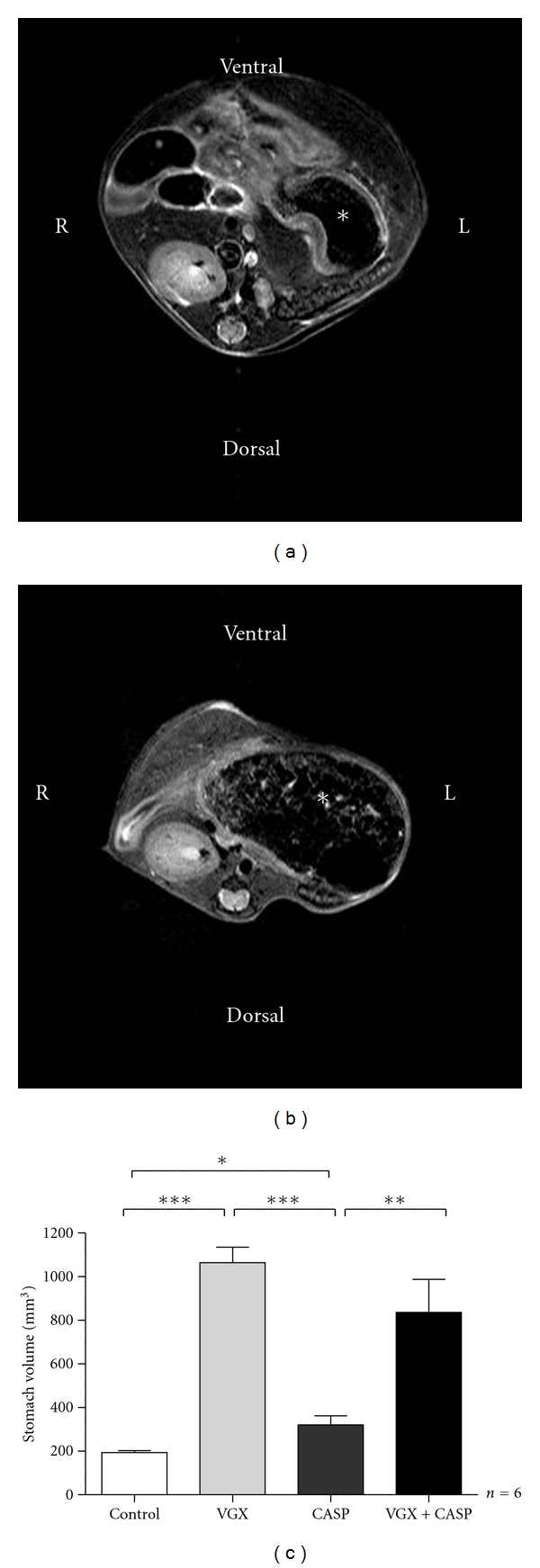
Images of MRI of the upper abdomen of mice. The asterisk identifies the stomach. Panel a shows the sectional image of an untreated mouse with normal gastric volume (a). Panel b displays the representative scan of a mouse with increased gastric volume after CASP and vagotomy (b). Values of gastric volume are shown in panel c. CASP alone led to a slight but significant increase of gastric volume. In both vagotomy groups, a strong dilatation of the stomach was measured that was independent from the presence of sepsis (c). (*n* = 6 per group, *P < 0.05, **P < 0.01, and ***P < 0.0001).

By visualization of the stomach in small animal 7-Tesla-MRI, we confirmed that vagotomy results in an increased gastric volume.  Figure 3(a) displays the regular empty gastric volume (193 ± 9 mm^3^) in untreated animals. Seven days following vagotomy, the volume was extended to 1064 ± 71.7 mm^3^. This difference is significant as shown in panel c (*n* = 6, *P* < 0.0001). In polymicrobial sepsis, the gastric volume was detected with 320.8 ± 41.64 mm^3^, which is a significant increase to the control group (*P* < 0.05) but a decrease as compared with the volume following the vagotomy procedure (*P* < 0.0001). Induction of peritonitis in vagotomised mice results in an enlarged gastric volume of 836.7 ± 151 mm^3^ at 24 hours postoperative. Vagotomy increases the gastric volume in the presence as well as in the absence of sepsis. These data suggest that the dilated intestine and the resulting ileus may not be the reason for the increased mortality in vagotomised septic mice, as these findings are also observed in septic mice (100% survival).

### 3.3. The Stimulation of Nicotinic Acetylcholine Receptors Has No Effect on the Survival in Polymicrobial Sepsis

**Figure 4:**
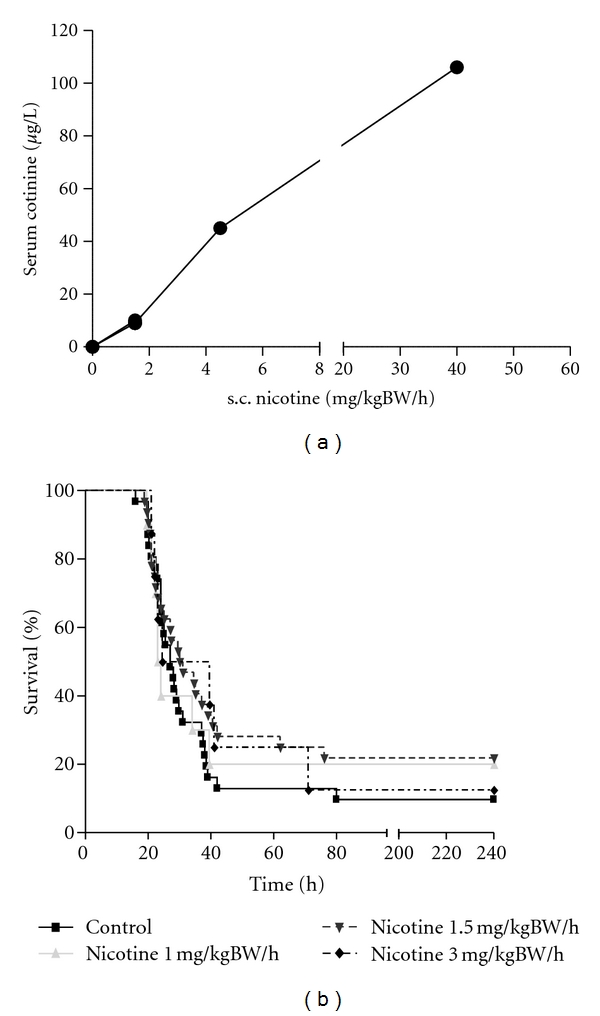
(a) To verify the therapeutic dose of nicotine in the murine serum, we analysed the serum cotinine level as this correlates with the amount of nicotine dose applicated by osmotic pumps. Application of nicotine in a dose of 1.5 mg/kgBW/h results in a serum cotinine level of 9.24 *μ*g/l (*n* = 4). 4.5 mg/kgBW/h nicotine leads to a cotinine serum concentration of 45 *μ*g/l (*n* = 1) and 40 mg/kgBW/h nicotine induces a cotinine serum concentration of 106 *μ*g/l (*n* = 1). Serum samples were analysed 18 hours following CASP. (b) The survival in CASP is not altered by continuous nicotine administration through subcutaneously implanted osmotic pumps. A nicotine exposition of 1 mg/kg bw per hour correlates with a survival rate of 20% (*n* = 10), 1.5 mg/kgBW/h (*n* = 32) nicotine leads to a survival rate of 21.85% and 3 mg/kgBW/h nicotine correlates with a survival rate of 12,5% (*n* = 8). In the control group, NaCl was applied where the survival rate was detected with 9.76% (*n* = 31).

The influence of a continuous application of nicotine on the survival in sepsis was analysed following implantation of osmotic pumps subcutaneously before induction of CASP. By this method, a detectable and dose-dependent serum level of cotinine, a metabolite of nicotine, can be reached (Figure 4(a)). The survival rate in septic peritonitis during continuous administration of nicotine in different body-weight (BW)-adapted dosages was compared: 1 mg/kgBW/h, 1.5 mg/kgBW/h, and 3 mg/kgBW/h. In the control group the osmotic pumps released NaCl 0.9%, and the survival rate was detected by 9.67% (Figure 4(b), *n* = 31). A dosage of 1 mg/kgBW/h correlates with a survival of 20% (*n* = 10), 1.5 mg/kgBW/h (*n* = 32) leads to a survival of 21.85% and 3 mg/kgBW/h to a survival of 12.5% (*n* = 8). These data suggest that a systemic application of the unspecific nicotinic acetylcholine receptor agonist nicotine has no protective effect on the outcome of septic peritonitis.

### 3.4. Serum Corticosterone Levels in Sepsis Are Significantly Elevated in Vagotomised Mice

**Figure 5:**
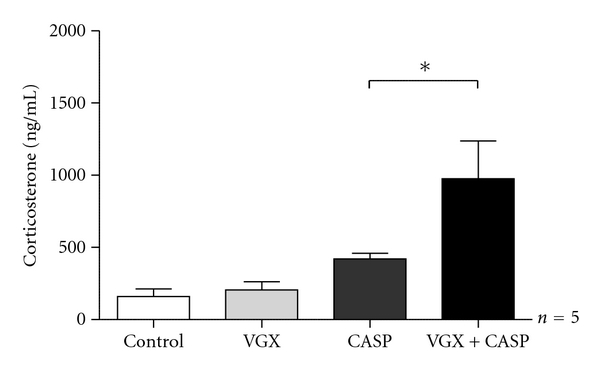
The serum levels of corticosterone in sepsis are modulated by the vagus nerve. Vagotomy in the nonseptic organism does not influence the corticosterone level (205.9 ± 56.75 ng/mL). In polymicrobial sepsis, serum corticosterone is detected with 159.4 ± 53.24 ng/mL. In contrast, 24 h following CASP in vagotomised mice the corticosterone level is significantly increased up to 975.4 ± 261.9 ng/mL (*P = 0.031, Mann-Whitney, *n* = 5).

Due to the central role assigned to adrenal glands in peritonitis [13–15, 35], we focused on the influence of the vagus nerve on the adrenal gland function in polymicrobial sepsis (Figure 5). Vagotomy in the nonseptic organism did not influence the corticosterone level (205.9 ± 56.75 ng/mL) as compared to a control group. In polymicrobial sepsis, the serum corticosterone was detected with 159.4 ± 53.24 ng/mL. In contrast, 24 h following CASP in vagotomised mice the corticosterone level is significantly increased up to 975.4 ± 261.9 ng/mL (*P = 0.031, *n* = 5). This indicates that the serum levels of corticosterone in sepsis are modulated by the vagus nerve.

### 3.5. The Vagus Nerve Has a Stimulating Effect on the *EX Vivo* Cytokine Release of Kupffer Cells

Kupffer cells were isolated seven days following vagotomy. The levels of TNF-*α* were detected in the cell culture supernatant. Kupffer cells isolated from untreated animals served as control. The basal TNF-*α* release of Kupffer cells *ex vivo* was significantly decreased as compared with the control group (164.7 ± 40.9 pg/mL versus 61.1 ± 4.4 pg/mL, *P = 0.04). Furthermore, we stimulated Kupffer cells *ex vivo* with 1 *μ*g/mL lipopolysaccharide from *E. coli* (LPS). In the septic organism, there was a significantly decreased TNF-*α* release by stimulated Kupffer cells from vagotomised mice (2960 ± 513.1 pg/mL) when compared to mice with an intact vagus nerve (5746 ± 292.5 pg/mL, ***P = 0.0002, *n* = 10 per group). These data substantiate the hypothesis that the vagus nerve has an immunological influence on Kupffer cell function.

## 4. Discussion

The present study underlines the crucial role of the vagus nerve for the survival in septic peritonitis. Impaired vagal function results in increased mortality rates in inflammatory animal models (CASP) [7]. Stimulation of nervus vagus function ameliorates survival as described for different animal models like CLP, LPS application, or i.p. *E. coli* injection [1, 6, 36]. Additionally, the vagus nerve has strong influence on the intestinal tonus: increased ileus incidence is described following vagotomy [37], whereas vagus stimulation attenuates the postoperative ileus [38].

Surgical trauma or peritoneal inflammation can result in paralysis and consecutive ileus, too. It could therefore be possible that the ileus triggered by vagotomy is the critical factor of decreased survival rate in CASP following vagotomy. Vagotomy itself causes an increased pylorus tone with delayed gastric emptying (DGE) as we could describe by measuring gastric volumes in MRI scans (Figure 3(a)). Vagotomy without induction of sepsis significantly enlarged gastric volume when compared to CASP mice. In combination of both, Vagotomy and CASP, the vagotomy effect seems to stimulate ileus by sepsis. Our data on postoperative mortality suggest an unchanged survival rate in vagus dissected animals (Figure 2). Surgical sectioning of the vagus nerve per se is performed in procedures like gastrectomy or oesophagectomy and itself does not result in elevated mortality rates [39–42]. Therefore, vagal impairment is responsible for marked DGE and ileus but not for higher mortality.

Vagotomy results in an attenuated release of nicotine in efferent signaling. The role of nicotine in sepsis, especially its possible therapeutic effect, and stimulation of the vagus nerve were subject of several studies: The et al. described reduced experimental postoperative Ileus [43] using a central stimulus for the cholinergic pathway. Other studies define better survival in CLP and LPS models using nicotine as an unspecific stimulator of nACHR [1, 36]. In our experiments, we decided for continuous nicotine administration by subcutaneously implanted osmotic pumps, since in our opinion permanent administration ensures sufficient serum levels, especially due to the short half life of nicotine in C57Bl/6 mice (about 9 minutes) [44]. We reached adequate doses as demonstrated by the serum level of the nicotine metabolite cotinine (Figure 4(a)). We observed no change in survival rates by administration of nicotine (Figure 4(b)). This observation may attribute to our CASP model which in contrast to CLP or LPS models is associated with a very high bacterial load [30, 32]. Our finding of worse survival correlates with Westerloo et al. who administered living *E. coli* i.p. in mice [6]. They also detected even decreased survival after oral nicotine substitution. Action potentials transmitted in the vagus nerve lead to release of acetylcholine that blocks cytokine production by cells-expressing acetylcholine receptors. In particular, the efferent vagus can inhibit inflammation via interaction between acetylcholine and *α*7 subunit of cholinergic receptors [45]. Signal transduction by the nicotinic *α*7 cholinergic receptor subunit is the regulator of intracellular signals that control cytokine transcription and translation. Neutrophils expressing several nicotinic receptors, including the *α*7 cholinergic receptor [23], and stimulation of these receptors have been shown to inhibit neutrophil migration by a mechanism that involves inhibition of adhesion molecule expression on both the endothelial surface and neutrophils [23]. Mice deficient in *α*7 cholinergic subunit have an optimized bacterial clearance caused by a faster recruitment of neutrophils [46]. As the early recruitment of neutrophils to the site of an infection is considered important for an adequate antibacterial defense our present results are consistent with reports showing that nicotine (which stimulates *α*7 receptors) facilitates the growth and dissemination of *E. coli *after intraperitoneal infection [6]–the protective effect of nicotine administration seems to be not potent enough in case of massive living pathogen load.

Another advice for higher mortality in polymicrobial sepsis are the significantly increased corticosterone levels in vagotomized mice (Figure 5). Especially human cortisol has several anti-inflammatory and immunosuppressive effects, that is, reduced TNF-alpha, increased IL-10-concentrations and apoptosis of mature T-lymphocytes [16, 47]. Adrenal insufficiency is frequently diagnosed in critical ill patients with sepsis [48] and it is associated with a high mortality rate [13–15]. A glucocorticoide administration during human sepsis was a discussed controversial [17, 18]. In rodents cortisol is replaced by corticosterone because of lack of C17-hydroxylase function [49, 50], in many ways [51]. We found increased corticosterone levels in septic animals that had undergone subdiaphragmal vagotomy seven days before CASP procedure (Figure 5). CASP mice with intact vagus nerve had a moderate but not significant elevation of serum corticosterone, whereas vagotomy alone had no effect on serum corticosterone levels.

To compensate for vagotomy, the endocrine axis not only seems to develop a stronger countereffect, as reflected by the corticosterone response [52]: Our data support the hypothesis of a relevant connection between vagus nerve and adrenal glands [10–12, 53] and suggest a vagal regulative function concerning hypoinflammation during sepsis via corticosterone.

In the course of sepsis immune system alternates between hyper- and hypo-inflammation. Increased proinflammatory TNF-levels and compensatory anti-inflammation with increased IL-10 levels contribute to immune paralysis status [54–56]. This effect seems to be dependent of vital pathogens, corticosterone responses were not affected in LPS models evaluating effects of vagotomy by Hansen et al. [57] or Gaykema et al. [58].

**Figure 6:**
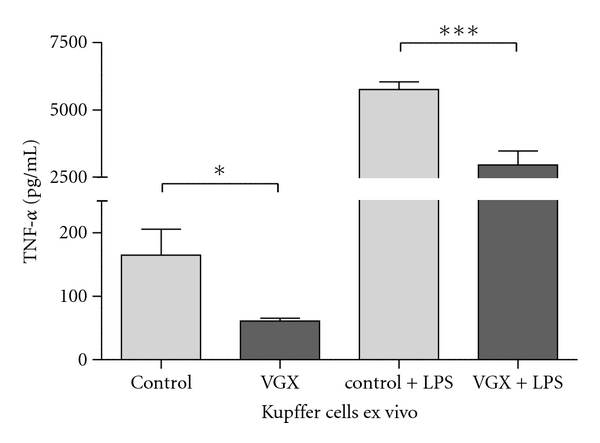
The vagus nerve has a stimulating effect on kupffer cells. Kupffer cells were isolated from control mice (column “control”) and from vagotomised mice without sepsis (column “VGX”). The basal TNF release of kupffer cells *ex vivo* is significantly decreased if mice were vagotomised 7 days before cell isolation (164.7 ± 40.9 pg/mL versus 61.1 ± 4.4 pg/mL, *P = 0.04). In addition, we stimulated the cells *ex vivo* with 1 *μ*g/mL lipopolysaccharide from *E.coli* (LPS). In the septic organism, there is a significantly decreased TNF-*α* release by stimulated kupffer cells from vagotomised mice (2960 ± 513.1 pg/mL) when compared to mice with an intact nervus vagus (5746 ± 292.5 pg/mL, ***P = 0.0002, *n* = 10 per group).

Furthermore, our data from in vitro experiments with Kupffer cells (KCs) indicate in the same line. KCs taken from vagotomized mice secreted reduced TNF-alpha amount after LPS stimulus (Figure 6).

Kupffer-cells are tissue macrophages located in the liver [27]. They liberate cytokines like TNF-alpha, IL-1, IL-6, and IL-10 and chemokines such as MCP-1 during inflammation [28]. Ikeda et al. stimulated Kupffer cells with acetylcholine and detected increased IL-6 secretion rates [23]. In our model, we performed subdiaphragmal vagotomy to dissect the established anatomical link by the hepatic branch of the vagus nerve [25, 26]. Our data correlate with Ikedas studies on cytokine release: *ex vivo* LPS stimulation induces a high level of TNF-alpha secretion. This LPS effect is attenuated in KCs isolated from animals that had undergone vagotomy seven days before (Figure 6). Basal rate (without LPS) of TNF-alpha secretion is significantly lower in vagotomy group. This underlines that the vagus nerve has a stimulating effect on the Kupffer cell activity. Following vagotomy, these macrophages are inhibited in their immunologic function, so the organism is impaired in its ability to cope with the septic situation. Their sensitivity seems to be downregulated resulting in less release of proinflammatory cytokines.

In summary, past studies have shown that the vagus nerve controls the immune response of hyperinflammation. Yet, our studies also suggest that the vagus nerve controls both stimulation as well as inhibition of inflammatory responses to severe bacterial threats. A high load of pathogens has unmasked the effects of the vagus nerve on states of hypoinflammation in our CASP model. Future studies will show the impact of further therapeutic modulation.

## 5. Conclusion

The vagal nerve plays an important role during peritonitis and leads to increased sepsis mortality after vagotomy. A stimulation of cholinergic receptors by nicotine has no therapeutic effect. Increased septic mortality seems to be a consequence of the absent modulating influence of the vagus nerve on the immune system. To underline this hypothesis we detect significantly elevated serum corticosterone levels in vagotomised mice 24 h following CASP and a decreased *ex vivo* TNF-alpha secretion of Kupffer cells upon stimulation with LPS. The recent study suggests that the vagus nerve controls both stimulation as well as inhibition of inflammatory responses to severe bacterial threats.
